# Retention in care among HIV-positive patients initiating second-line antiretroviral therapy: a retrospective study from an Ethiopian public hospital clinic

**DOI:** 10.3402/gha.v9.29943

**Published:** 2016-01-12

**Authors:** Sten Wilhelmson, Anton Reepalu, Taye Tolera Balcha, Godana Jarso, Per Björkman

**Affiliations:** 1 Section for Infectious Diseases, Institution of Clinical Sciences, Lund University, Malmö, Sweden; 2 Ministry of Health of Ethiopia, Addis Abeba, Ethiopia; 3 Adama Regional Hospital, Adama, Ethiopia

**Keywords:** HIV, second-line, antiretroviral therapy, loss to follow-up, Ethiopia, sub-Saharan Africa, retention in care

## Abstract

**Background:**

Access to second-line antiretroviral therapy (ART) for HIV-positive patients remains limited in sub-Saharan Africa. Furthermore, outcomes of second-line ART may be compromised by mortality and loss to follow-up (LTFU).

**Objective:**

To determine retention in care among patients receiving second-line ART in a public hospital in Ethiopia, and to investigate factors associated with LTFU among adults and adolescents.

**Design:**

HIV-positive persons with documented change of first-line ART to a second-line regimen were retrospectively identified from hospital registers, and data were collected at the time of treatment change and subsequent clinic visits. Baseline variables for adults and adolescents were analyzed using multivariate Cox proportional hazards models comparing subjects remaining in care and those LTFU (defined as a missed appointment of ≥90 days).

**Results:**

A total of 383 persons had started second-line ART (330 adults/adolescents; 53 children) and were followed for a median of 22.2 months (the total follow-up time was 906 person years). At the end of study follow-up, 80.5% of patients remained in care (adults and adolescents 79.8%; children 85.7%). In multivariate analysis, LTFU among adults and adolescents was associated with a baseline CD4 cell count <100 cells/mm^3^ and a first-line regimen failure that was not confirmed by HIV RNA testing.

**Conclusions:**

Although retention in care during second-line ART in this cohort was satisfactory, and similar to that reported from first-line ART programs in Ethiopia, our findings suggest the benefit of earlier recognition of patients with first-line ART failure and confirmation of suspected treatment failure by viral load testing.

Antiretroviral therapy (ART) has radically changed the prognosis for people living with HIV (PLHIV). In the past decade, access to ART has increased worldwide, and in 2014, 14.8 million people had initiated ART globally (1). This increase has been particularly strong in sub-Saharan Africa, the region of the world region where the majority of PLHIV reside. World Health Organization (WHO) guidelines recommend standardized first-line regimens based on non-nucleoside reverse transcriptase inhibitors (NNRTIs; 2). Overall outcomes of first-line regimens in low-income countries have been found to be satisfactory and similar to those in high-resource settings (3). Yet, a growing number of patients are expected to experience treatment failure over time, with a subsequent escalating need for second-line regimens (4, 5). Routine virological monitoring for treatment response is not available in most ART programs in low-income countries, in contrast to high-income countries. Instead, first-line ART failure is defined by clinical and/or immunological criteria (2). In the absence of regular viral load testing, the recognition of persons with treatment failure is often delayed (6, 7). As a consequence, selection of extensive antiretroviral drug resistance may have occurred before treatment failure is identified (8, 9), compromising outcomes of subsequent antiretroviral regimens with regard to virological suppression (10). Conversely, many individuals who meet the criteria for clinical or immunological ART failure have been shown to have undetectable HIV viremia. These findings are valid for both adults and children (11–13).

Irregular drug intake is an important cause of treatment failure. Hence it is possible that patients with first-line failure due to inadequate drug adherence are at high risk of a subsequent treatment failure on second-line ART (14). Irregular drug intake exists for many reasons. Ensuring adequate retention in care is one major challenge for ART programs in resource-limited settings. In a review based on 39 cohorts in sub-Saharan Africa, nearly one- third of patients did not remain in care 3 years after starting ART, with loss to follow-up (LTFU) accounting for 59% of the cases and death for 41% of the cases (15). Irregular treatment has been associated with increased mortality and morbidity, as well as with virological failure and the selection of HIV drug resistance (16, 17).

In Ethiopia, a free public ART program was introduced in 2005 and since then the number of persons starting ART has steadily increased. In 2014, the number of persons initiated on ART treatment was 362,000 – equal to a treatment coverage of 61% (1, 18). As a consequence, both HIV-related mortality and HIV incidence have decreased in the country (1, 18, 19). The number of patients on second-line ART remains low in Ethiopia; 1.7% of all ART recipients. The low number reflects difficulties in the recognition of subjects eligible for second-line therapy as well as restricted access to such treatment, a predicament Ethiopia shares with many other sub-Saharan African countries (20). In order to optimize second-line ART programs, it is important to understand the outcome of such therapy and identify predictors of adverse treatment outcomes. In this retrospective study, we have determined the retention in care among patients initiating second-line ART in a large public ART clinic in Ethiopia and investigated the factors in the switch to second-line ART that are associated with subsequent LTFU in adults and adolescents.

## Methods

### Setting

The study was conducted at the outpatient ART clinic at Adama Regional Hospital. Adama, the economic capital of the Oromia region, is located in Central Ethiopia, and has an estimated population of 300,000 inhabitants. This ART service was introduced in 2005 and is considered to be the second largest HIV clinic in Ethiopia. HIV care, including ART initiation and monitoring, is provided by specially trained nurses who follow national guidelines. Physicians are available for consultation and are responsible for the switch of therapy to second-line regimens. Clinical and/or immunological criteria (the occurrence of new HIV-related conditions and the absence of CD4 cell response, respectively, as defined by WHO) (2), are used for the identification of suspected first-line ART failure. Since 2010, suspected treatment failure has been routinely confirmed by HIV RNA determination. Second-line regimens are prescribed following recommendations in national HIV guidelines, which involves the replacement of the NNRTI component with a protease inhibitor (PI), and with the switch of one or both nucleos(t)ides (NRTI) from the first-line regimen. Drug resistance testing for selection of antiretroviral drugs for second-line ART is not available.

### Study design

All persons who, according to hospital registers, had initiated second-line ART before July 27, 2014 (3 months before the collection of data) were eligible for inclusion. Second-line ART was defined as a modification of a first-line ART regimen to a PI-containing regimen and could occur either due to first-line treatment failure or because of side effects to first-line antiretroviral drugs. Patients who were alive at the time of data collection and who did not meet criteria for LTFU were considered to remain in care. Persons with the documented transfer of care to another health facility were not included in this analysis. LTFU was defined as having missed a scheduled appointment by 90 days or more. Subjects of all ages were eligible for inclusion, but for the analysis of factors associated with LTFU, only adults and adolescents (defined as persons aged 15 years or greater) were included.

Participant data was collected retrospectively from ART registers at the clinic until any of the following events: death, transfer of care, LTFU, or up to 3 months before the start of data collection. Characteristics recorded at the last visit preceding the initiation of second-line ART were used as baseline variables.

### Data collection and analysis

According to the standard procedure at the clinic, medical information is obtained by health workers at each clinic visit following standardized questionnaires (including detailed socio-economic data at the first clinic visit), and entered into patient cards. Information from patient cards is transferred to an electronic register at regular intervals. Data for this study was primarily collected from patient cards, whereas an electronic register was used when information from patient cards could not be retrieved. Data on antiretroviral drug combinations and HIV RNA were also collected from prevailing pharmacy and laboratory registers, respectively. Study data was entered into a research database by one of the authors (SW; October to December 2014), using subject-specific study codes.

The primary outcome was retention in care after the initiation of second-line ART. For the analysis of risk factors for LTFU only adults and adolescents were included. Adults and adolescents remaining in care were compared to those meeting the criteria for LTFU. Patients with documented mortality or transfer of care were excluded from this analysis. The following variables were selected and included for analysis of risk factors for LTFU: age, gender, CD4 cell count strata, WHO clinical stage, the performance of viral load testing prior to regimen change, functional status, education, marital status, access to household electricity, and access to a household water supply. Time-at-risk was defined as the number of days on a second-line regimen at the clinic before a study end-point. The Mann-Whitney U test was used to compare CD4 cell counts between patients LTFU and patients still in care. Associations between baseline factors and LTFU were first analyzed in a univariate analysis. Factors with *p*<0.3 in univariate analysis were further analyzed in a multivariate Cox model, using backward step-by-step exclusion, removing the least significant variable, until all remaining variables had a *p*<0.05. Kaplan-Meier curves were used to ensure that all variables included in the model fulfilled the proportional hazards assumption.

SPSS for Windows version 22 was used for all statistical analyses.

### Ethical approval

Ethical approval was granted from the Institutional Review Board at the Oromia Regional Health Bureau. Due to the retrospective study design and its objective to analyze LTFU, individual informed consent was not sought. To ensure confidentiality, all study data was managed under code.

## Results

### Study participants

Among 427 eligible patients, 383 subjects were included (330 adults/adolescents, 53 children). Among the excluded 44 persons, 25 were excluded due to missing data (mainly persons who had initiated second-line ART in other clinics), and 19 were excluded because they had initiated PI-based first-line ART (these were mainly children who had been exposed to NNRTI *in uteri*). At the initiation of second-line ART, the median age was 36.6 years (interquartile range [IQR] 30.0–42.0) for adults/adolescents and 11.2 years (IQR 7.3–13.8) for children. The median CD4 cell count was 126 cells/mm^3^ (IQR 65.5–226.0) for adults/adolescents and 154 cells/mm^3^(IQR 88–319) for children. The baseline characteristics of these subjects are shown in Tables 1 and 2.

**Table 1:** Characteristics of adults and adolescents^a^ at initiation of second-line ART with regard to subsequent retention in care

	Total, *n* (%) (*N*=330)	Retention in care, *n* (%) (*n*=267)	No retention in care, *n* (%) (*n*=63)
Age category (Years)			
15–29	102 (31.0)	85 (31.8)	17 (27.0)
30–39	141 (42.7)	113 (42.3)	28 (44.4)
≥ 40	87 (26.4)	69 (25.8)	18 (28.6)
Gender			
Female	180 (54.5)	142 (53.2)	38 (60.3)
Male	150 (45.5)	125 (46.8)	25 (39.7)
CD4 category (cells/mm^3^)			
≥ 100	192 (58.2)	161 (60.3)	31 (49.2)
< 100	131 (39.7)	101 (37.8)	30 (47.6)
N/A	7 (2.1)	5 (1.9)	2 (3.2)
WHO stage category			
Stage 1–2	109 (33.0)	97 (36.3)	12 (19.0)
Stage 3–4	216 (65.5)	166 (62.2)	50 (79.4)
N/A	5 (1.5)	4 (1.5)	1 (1.6)
Functional status			
Able to work	261 (79.1)	220 (82.4)	41 (65.1)
Unable to work	65 (19.7)	43 (16.1)	22 (34.9)
N/A	4 (1.2)	4 (1.5)	0 (0.0)
First-line ART failure confirmed by HIV RNA testing			
Yes	178 (53.9)	162 (60.7)	16 (25.4)
No	141 (42.7)	98 (36.7)	43 (68.3)
N/A	11 (3.3)	7 (2.6)	4 (6.3)
Reasons for switch to second-line ART			
Treatment failure	216 (65.5)	187 (70.0)	29 (46.0)
Side effect	74 (22.4)	58 (21.7)	16 (25.4)
Other	6 (1.8)	5 (1.9)	1 (1.6)
N/A	34 (10.3)	17 (6.4)	17 (27.0)
Education			
No education	58 (17.6)	43 (16.1)	15 (23.8)
Primary education or higher	268 (81.2)	221 (82.8)	47 (74.6)
N/A	4 (1.2)	3 (1.1)	1 (1.6)
Marital status			
Married	145 (43.9)	117 (43.8)	28 (44.4)
Not married	181 (54.8)	147 (55.1)	34 (54.0)
N/A	4 (1.2)	3 (1.1)	1 (1.6)
Running water			
Yes	171 (51.8)	138 (51.7)	33 (52.4)
No	133 (40.3)	109 (40.8)	24 (38.1)
N/A	26 (7.9)	20 (7.5)	6 (9.5)
Electricity			
Yes	230 (69.7)	188 (70.4)	42 (66.7)
No	74 (22.4)	59 (22.1)	15 (23.8)
N/A	26 (7.9)	20 (7.5)	6 (9.5)

ART, antiretroviral treatment

a Nine patients with documented transfer of care were excluded.

**Table 2:** Characteristics of children (age<15 years)^a^ at initiation of second-line ART with regard to subsequent retention in care

	Total, *n* (%) (*N*=53)	Retention in care, *n* (%) (*n*=46)	No retention in care, *n* (%) *(n*=7)
Age (years)			
≤ 7	27 (50.9)	24 (52.2)	3 (42.9)
> 7	26 (49.1)	22 (47.8)	4 (57.1)
N/A	0 (0.0)	0 (0.0)	0 (0.0)
Gender			
Male	25 (47.2)	24 (52.2)	1 (14.3)
Female	27 (50.9)	21 (45.7)	6 (85.7)
N/A	1 (1.9)	1 (2.2)	0 (0.0)
CD4 category (cells/mm^3^)			
< 200	30 (56.6)	26 (56.5)	4 (57.1)
> 200	19 (35.8)	17 (37.0)	2 (28.6)
N/A	4 (7.5)	3 (6.5)	1 (14.3)
BMI (kg/m^2^)			
< 15	22 (41.5)	20 (43.5)	2 (28.6)
> 15	26 (49.1)	22 (47.8)	4 (57.1)
N/A	5 (9.4)	4 (8.7)	1 (14.3)
First-line ART failure confirmed by HIV RNA testing			
Yes	37 (69.8)	35 (76.1)	2 (28.6)
No	15 (28.3)	10 (21.7)	5 (71.4)
N/A	1 (1.9)	1 (2.2)	0 (0.0)
Reasons for switch to second-line ART			
Treatment failure	37 (69.8)	35 (76.1)	2 (28.6)
Side effect	14 (26.4)	10 (21.7)	4 (57.1)
Other	1 (1.9)	0 (0.0)	1 (14.3)
N/A	1 (1.9)	1 (2.2)	0 (0.0)
At least one parent alive			
Yes	36 (67.9)	31 (67.4)	5 (71.4)
No	6 (11.3)	5 (10.9)	1 (14.3)
N/A	11 (20.8)	10 (21.7)	1 (14.3)

ART, antiretroviral treatment; BMI, body mass index.

a Four patients with documented transfer of care were excluded.

### Indications for second-line ART

#### Adults/adolescents

The indication for second-line ART was treatment failure in 216 (65.5%) cases, side effects on first-line ART in 74 (22.4%) cases, and other stated reasons in 6 (1.8%) cases. In 34 (10.3%) cases, the reason was not stated. Among the 216 subjects with treatment failure, 182 (84.3%) fulfilled immunological criteria, and 71 (32.9%) fulfilled clinical criteria. Treatment failure was confirmed by viral load testing in 182 (84.3%) subjects. At the time of treatment switch, the median viral load was 72,900 copies/mL (IQR 29,350–220,500; >10,000 copies/mL in 164 [92%] cases).

#### Children

Thirty-seven subjects (69.8%) changed to a second-line regimen due to treatment failure, 14 (26.4%) changed due to side effects on a first-line regimen, 1 (1.9%) due to initiation of tuberculosis treatment, and 1 (1.9%) for unknown reasons. Among the 37 children with first-line treatment failure 32 (86.5%) fulfilled immunological criteria, and 14 (37.8%) fulfilled clinical criteria. Treatment failure was confirmed by viral load testing in all 37 subjects. The median viral load was 92,300 copies/mL (IQR 38,200–323,500; >10,000 copies/ml in 35 [95%] cases).

### Retention in care

Overall retention in care was 80.5%, whereas 18.9% met criteria for LTFU.

#### Adults/adolescents

At the end of follow-up, 256/321 (79.8%) patients remained in care of the total 330. The nine patients unaccounted for were censored due to recorded transfer of care to another health facility. Two (0.6%) were confirmed dead, and 63 (19.6%) met the definition of LTFU. Among the 63 patients LTFU, nine returned to care at the study clinic during the follow-up period. The 330 adults/adolescents were followed for a median of 22.1 months (783 person years). For patients who were LTFU, the median follow-up time was 8.7 months. The CD4 cell count at the last follow-up visit was higher for patients who remained in care (median 429 cells/mm^3^ [IQR 261–607]), compared to subjects LTFU (median 209 cells/mm^3^ [IQR 68–369]), *p*<0.01.

#### Children

At the end of follow-up, 42/49 (85.7%) patients remained in care of the total 53. The four patients unaccounted for were censored due to recorded transfer of care to another health facility. None were confirmed dead, and 7 (14.3%) met the definition of LTFU. Among those LTFU, three returned to care at the study clinic during the follow-up period. The 53 children were followed for a median of 23.5 months (123 person years). For the subjects who were LTFU, the median time on second-line ART was 6.4 months. The CD4 cell count at the last follow-up visit was higher for subjects who remained in care (median 673 cells/mm^3^ [IQR 433–785]) than for the patients who were LTFU (median 374 cells/mm^3^ [IQR 210–826]), but this difference did not reach statistical significance (*p=*0.22).

### Risk factors for LTFU during second-line ART in adults and adolescents

In univariate Cox analysis, the following variables showed an association with LTFU (*p*<0.3): CD4 cell count <100 cells/mm^3^, WHO status 3 or 4, being unable to work, having no education, and first-line ART failure not confirmed by viral load testing (Table 3). In the multivariate Cox model, LTFU was independently and significantly associated with a CD4 cell count <100 cells/mm^3^at the time of the switch to second-line ART (HR 2.24; 95% CI: 1.31, 3.84), and first-line ART failure not confirmed by viral load testing (HR 2.50; 95% CI: 1.34, 4.66) (Table 3).

**Table 3:** Cox proportional hazards model for LTFU for adults and adolescents

			Multivariate			
			
	Univariate		Before step-by-step exclusion		After step-by-step exclusion	
			
Variable	HR (95% CI)	*p*	HR (95% CI)	*p*	HR (95% CI)	*p*
Age category (years)						
15–29	1.0					
30–39	0.9 (0.46–1.76)	0.764				
> 40	1.03 (0.57–1.86)	0.933				
Gender						
Female	1.0					
Male	1.15 (0.69–1.91)	0.596				
CD4 category (cells/mm^3^)						
≥ 100	1.0		1.0		1.0	
< 100	1.93 (1.16–3.20)	0.012*	2.36 (1.36–4.09)	0.002**	2.24 (1.31–3.84)	0.003**
WHO stage category						
Stage 1–2^a^	1.0		1.0			
Stage 3–4	1.51 (0.80–2.86)	0.205*	1.02 (0.51–2.04)	0.952		
Functional Status						
Able to Work	1.0		1.0			
Unable to Work	1.97 (1.17–3.33)	0.011*	1.59 (0.89–2.85)	0.119		
First-line failure confirmed by HIV RNA testing						
Yes	1.0		1.0		1.0	
No	2.11 (1.16–3.83)	0.014*	2.08 (1.09–3.98)	0.026**	2.50 (1.34–4.66)	0.004**
Education						
Primary education or higher	1.0		1.0			
No education	1.74 (0.97–3.13)	0.064*	1.58 (0.83–2.97)	0.161		
Marital status						
Not married	1.0					
Married	1.05 (0.64–1.74)	0.848				
Running water						
No	1.0					
Yes	1.22 (0.72–2.07)	0.453				
Electricity						
No	1.0					
Yes	1.06 (0.58–1.91)	0.859				

HR, hazard ratio; CI, confidence interval.

* Variables with *p*<0.3 in univariate analysis were included in the multivariate model. In the multivariate model a backward step-by-step exclusion Cox proportional hazards model was used.

** *p*<0.05 were considered significant.

a According to clinical staging for severity of HIV disease (21).

## Discussion

Among patients starting second-line ART at a hospital-based clinic in Ethiopia, 80.5% remained in care after a median of 22.2 months of follow-up, a finding that supports the continued and expanded provision of second-line ART in the Ethiopian public health system. The main cause of attrition among both adults and adolescents and children was LTFU after starting second-line ART, which occurred in 18.9% of study subjects. In adults and adolescents, LTFU was associated with CD4 cell counts <100 cells/mm^3^ at the time of the switch in regimen (HR 2.24; 95% CI: 1.31, 3.84) and with the absence of viral load confirmation of first-line treatment failure (HR 2.50; 95% CI: 1.34, 4.66).

The rate of LTFU on second-line ART in our population is slightly higher compared to data from other second-line ART programs in sub-Saharan Africa and Asia, reporting proportions of LTFU ranging from 3.6 to 12% at 6 months (10, 22), 3.0–17% at 12 months (23–25), and 3.0–8.0% at 24 months (26, 27), respectively. The slightly higher rate of LTFU may be a general Ethiopian phenomenon, regardless whether patients are in first-line or second-line ART. Several studies have previously shown high rates of LTFU among both adults and adolescents, and children starting first-line ART in Ethiopia (28–31). Unpublished data, collected at the clinic where this study was conducted, indicates a LTFU rate of 28% among adults and adolescents on first-line ART. Another study from the same patient up-take area reports that 34% of the children on first-line ART were LTFU (31). Hence it appears that retention in care during the second-line ART, in this study, was not worse than that in Ethiopian patients on first-line regimens.

The uncertainty of classifying true outcomes is an inherent problem of studies on LTFU. Different explanations for LTFU in ART programs in sub-Saharan Africa have been described (32–34). In a district of Southern Ethiopia, the tracing of patients with LTFU revealed that 48% had died. In other cases, patients had registered for ART care at other facilities or resorted to alternative therapies (33). The low mortality rate (0.5%) in our study is likely an underestimate, and may reflect inadequately recorded mortality. The association between LTFU and indicators of advanced disease (such as low CD4 cell counts at the time of treatment switch and poor CD4 cell count increase at the last follow-up visit) suggest that unrecognized mortality explains a proportion of LTFU among our participants. However, the fact that a number of LTFU subjects did return to the clinic and resumed care during the study period shows that there are different explanations for LTFU. The second-line ART recipients lost to follow-up must be traced to gain a better understanding of the underlying mechanisms.

As is true for most ART programs in low-income countries, HIV RNA testing is not available for the routine monitoring of patients on first-line ART, and the identification of treatment failure still mainly depends on clinical and immunological criteria. In addition to the poor sensitivity of these criteria for detecting patients with viremia during ART, some patients with immunological and/or clinical signs of treatment failure have suppressed HIV RNA replication. In these cases, other medical conditions, in particular opportunistic infections such as tuberculosis, may be present; the modification of ART will not benefit such individuals. Since the introduction of HIV RNA testing in patients considered for second-line ART at the study clinic no patient with suspected treatment failure switched regimen in case of undetectable viremia. Routine HIV RNA testing for patients on first-line ART (irrespective of clinical or immunological characteristics) has been shown to identify treatment failure in its earlier stages, leading to less delay in the switch to second-line regimens (35). In addition, viral load testing has been shown to be a useful tool for improving and maintaining adherence (36).

Studies from first-line ART programs in Ethiopia have found various factors to be associated with subsequent LTFU, namely, characteristics related to medical and socio-economic condition, as well as the type of health care facility (37, 38). The same pattern has been observed among children starting first-line ART in Ethiopia (31). Factors associated with LTFU during second-line ART have hitherto not been studied. However, in findings similar to ours, advanced disease at treatment initiation has been found to be linked to the increased risk of death and treatment failure in adults starting second-line ART in other settings (20, 23). In addition, higher age and viral load at the time of the regimen switch have been associated with treatment failure in adults starting second-line ART (39).

The results of our study, performed in patients treated at a hospital-based public ART clinic in sub-Saharan Africa, suggest that earlier recognition of first-line treatment failure and subsequent regimen switch could lead to improved outcomes of second-line ART. Emphasis should be placed on expanding access to viral load testing in HIV programs, training ART prescribers how to recognize suspected treatment failure, and improving systems for tracing patients with LTFU. Other interventions may also be needed, such as the improved diagnosis of incident opportunistic infections and socio-economic support (35).

### Limitations

This study was based on retrospectively collected register data. However, patient information at this clinic has been collected and registered following structured questionnaires, and the proportion of missing data was low. The size of the study population was limited; with a larger number of participants, additional factors associated with LTFU could possibly have been identified. Since the number of children on second-line ART was low, and mechanisms of LTFU are likely to be different from those in adults and adolescents, we did not analyze the factors associated with LTFU in children.

Although the majority of participants had started second-line ART as a result of confirmed or suspected first-line treatment failure, we also included persons who had had treatment modification for other reasons. It is possible that retention in care, as well as factors associated with LTFU, differ with regard to the reason for changing to second-line ART. However, we did not find that indications for starting second-line ART were associated with LTFU.

## Conclusions

Retention in care during second-line ART at this Ethiopian clinic was satisfactory for both adults and adolescents and children. LTFU among adults and adolescents was associated with advanced immunosuppression at the time of ART regimen switch, and first-line treatment failure not confirmed by HIV RNA testing. These findings support the continued provision of second-line ART in the Ethiopian health system, with the confirmation of suspected treatment failure by viral load testing. This will require increased access to both virological monitoring and second-line antiretroviral drugs, as well as interventions to improve retention in care. Earlier recognition of first-line failure and the switch to second-line regimen before advanced immunosuppression has developed are likely to improve the outcomes of second-line ART.
